# The Relationship between Air Pollution and Brain Cancer: A Systematic Review and Meta-Analysis

**DOI:** 10.5334/aogh.3889

**Published:** 2023-06-23

**Authors:** Soheil Hassanipour, Hossein-Ali Nikbakht, Abdeltif Amrane, Morteza Arab-Zozani, Layla Shojaie, Saeid Rostami, Ahmad Badeenezhad

**Affiliations:** 1 Gastrointestinal and Liver Diseases Research Center, Guilan University of Medical Sciences, Rasht, Iran; 2 Social Determinants of Health Research Center, Health Research Institute, Babol University of Medical Sciences, Babol, Iran; 3 Univ Rennes, Ecole Nationale Supérieure de Chimie de Rennes, CNRS, ISCR-UMR 6226, F-35000 Rennes, France; 4 Social Determinants of Health Research Center, Birjand University of Medical Sciences, Birjand, Iran; 5 Division of GI/Liver, Department of Medicine, Keck school of Medicine, University of Southern California, Los Angeles, CA, USA; 6 Department of Environmental Health Engineering, Shiraz University of Medical Science, Shiraz, Iran; 7 Department of Environmental Health Engineering, School of Medical Sciences, Behbahan Faculty of Medical Sciences, Behbahan, Iran

**Keywords:** brain cancer, air pollution, systematic review, meta-analysis

## Abstract

**Background:**

There is very little epidemiological evidence on the effects of ambient air pollution on brain tumor risk. The purpose of this study was to determine the relationship between exposure to air pollution and the incidence of brain tumors.

**Methods:**

A comprehensive literature search in five international databases, including PubMed/Medline, ProQuest, Scopus, Embase, and ISI/WOS on April 15, 2019, was conducted. The methodology of the present study was based on the PRISMA (Preferred Reporting Items for Systematic Reviews and Meta-Analysis) statement. The Newcastle-Ottawa Quality Assessment Form was used to evaluate the quality of the selected papers.

**Results:**

Five studies that measured adult brain tumors as well as their long-term exposure to at least one of the pollutants criteria for air pollution, PM_2.5_ absorbance, and proximity to traffic (Trafnear) were reviewed. The results showed that the pooled relative risk (RR) for incidence of brain tumor and long term exposure to Trafnear, PM_2.5_, PM_2.5_ absorbance, O_3_ and NOx were RR = 1.07, (95% CI 0.99–1.16), P = 0.079, for Trafnear; RR = 0.90, (95% CI 0.80–1.00), P = 0.064 for PM_2.5_; RR = 1.63, (95% CI 1.04–2.55), P = 0.031 for PM_2.5_ absorbance; RR = 1.3, (95% CI 1.03–1.6), P = 0.023 for O_3_; and RR = 1.16, (95% CI 0.93–1.45), P = 0.173 for NOx. Exposure to other air pollutants had no statistically significant association with brain tumor incidence.

**Conclusion:**

The results showed that exposure to air pollutants, such as O_3_ and PM_2.5_ absorbance, had the highest correlation with brain tumor incidence. They also showed an absence of correlation between exposure to certain pollutants (SO_2_, CO, NO_2_, PM_10_, PM_2.5_) and brain tumor incidence.

## Introduction

The brain tumors are a class of neoplasms, which form diverse morphological subgroups with distinct behavior patterns. Nervous system cancers account for about 3% of all cancers in the world and are more common among men than women [1,2,3]. In general, the incidence of brain tumors is more leading into the west than in the east and more eminent in developed countries than in developing countries [4]. Air pollution is a blend of solid particles and gases in the air. Among the particulate components, particles smaller than 10 and 2.5 micrometers are of great importance for health [5]. These particles, along with gaseous compounds such as carbon monoxide (CO), nitrogen dioxide (NO_2_), ozone (O_3_) and sulfur dioxide (SO_2_), are known as standard pollutants of air pollution, especially in cities [6]. The primary sources of these pollutants include industry, automobile exhaust, forest fires, fixed fuel burners, and solid fuel combustion [7,8,9].

Nowadays, air pollution is a severe difficulty in numerous parts of the world and is acknowledged as one of the most hazardous environmental concerns [10,11]. Exposure to air pollution has numerous health effects, and due to the growing trend of pollutants, it threatens human health in many parts of the world [12,13,14,15]. Air pollutants can be a common source of inflammation, oxidative stress and DNA damage in humans [16,17]. The central nervous system is one of the systems affected by the impacts of air pollution [18,19].

Outdoor air pollution caused more than 3.2 million premature deaths globally [20]. Combustion of fossil fuels (coal, oil, and natural gas) contributed to an estimated one million deaths globally (27.3% of all mortality) [21]. Environmental pollutants may affect the central nervous system through several mechanisms, the transmission of gaseous pollutants and particles directly from the sinuses to the brain tissue through the olfactory nerves, the passage of pollutants through the alveoli, the inner wall of the lung tissue into the circulatory system, and the passage from the blood-brain barrier and creating a systemic inflammatory response in the lungs that may cause the release of oxygen free radicals in distant tissues such as the central nervous system [16,22]. In this regard, experimental evidence has shown that in animals, particle matter and O_3_ can reach the brain through inhalation or directly through the nose and the olfactory nerve, causing inflammation and destruction of nerve tissue [23]. While several types of cells in the brain react to pollutants, the latest reports suggest that microglia and brain capillaries may play a critical part in cellular damage [24]. The level of risk exhibited by these chemical and toxic compounds depends on the severity of exposure and biochemical metabolism in the brain [25].

Inflammation has been suggested to be very important in the pathogenesis of brain cancer. Also, genetic damage was observed in mice exposed to airborne particles that led to brain cancer [23]. Studies have shown that the progression of brain cancer is a multistage process that begins with genetic changes and damage to precancerous cells. However, there is little epidemiological evidence on the effects of air pollutants on brain cancer [26].

In this study, we aimed to examine the effects of air pollution criteria exposure in urban regions on the incidence of the central nervous system (CNS) cancer utilizing scientific proof, by reviewing prior studies through a systematic review and meta-analysis.

## Methods

The present study is a systematic review and meta-analysis of the relationship between exposure to air pollution and the incidence of brain tumors. This study was designed in 2019, and it is consistent with the PRISMA (Preferred Reporting Items for Systematic Reviews and Meta-Analysis) checklist [27].

### Search strategy

Five international databases including Medline/PubMed, ProQuest, Scopus, Embase, ISI/WOS with combinations of keyword including PM_2.5_, PM_10_ Coarse particles, Ozone (O_3_), Sulfur Dioxide (SO_2_) Nitrogen dioxide (NO_2_), Carbon Monoxide (CO), NOx, Trafnear and Brain Neoplasms were searched by researchers on April 15, 2019. All searches were entered into EndNote X7 software, and duplicate articles were automatically deleted. Two researchers examined the papers individually. There was no time period limit in the quest for studies.

### Inclusion and exclusion criteria

The studies that were considered, were as follows: 1) all cohort or longitudinal studies that address all or one of the benchmark pollutants including particles less than 10 µm in diameter (PM_10_), less than 2.5 µm (PM_2.5_), or between 2.5 and 10 microns (PM_2.5–10_), nitrogen oxides (NOx) or nitrogen dioxide (NO_2_), sulfur dioxide (SO_2_), ozone (O_3_), Trafnear and carbon monoxide (CO_2_); 2) the examined population included adults with brain cancer; and, 3) all studies were written in english. It is necessary to clarify that the references of the articles were also reviewed in order to add relevant studies.

Exclusion criteria included: (1) studies examining the exposure of these pollutants to animals; (2) studies that have examined exposure to tobacco smoke such as cigarette smoke; (3) occupational exposure studies; (4) studies for which there was no access to the full text even after the researchers’ follow-up (contacting authors by email).

### Quality assessment

The Newcastle-Ottawa Quality Assessment Form was used to evaluate the quality of the selected papers. This tool has three different parts including selection (four questions), comparability (one question) and outcome (three questions); and based on the final scores divided into three categories, good (three or four stars in selection domain, one or two stars in comparability domain and two or three stars in outcome/exposure domain), fair (two stars in selection domain, one or two stars in comparability domain and two or three stars in outcome/exposure domain) and poor (zero or one star in selection domain, 0 stars in comparability domain or 0 or 1 stars in outcome/exposure domain) [28] The results of the quality assessment are presented in appendix 1. Egger’s test was utilized to evaluate the risk of publication bias.

### Screening of studies

The initial search was conducted by two researchers (SH and HAN). Screening studies, extraction of results, and evaluation of the quality control of articles were performed individually (AB and HAN).In case of any disagreement, the team leader (SH) would make a final comment on the article.

### Statistical analysis

The heterogeneity amongst the included studies was assessed by the cochran test (with a significance level of less than 0.1) and its combination using I^2^ statistics (with a significance level greater than 50%). In the case of heterogeneity, the random-effect model was used with the inverse variance method, and in cases without heterogeneity, the fixed-effect model was employed. Because of the different indices published in the studies, the converted indices obtained by CMA software were used. The index used in this study was the risk ratio (RR). If the RR is 1 (or close to 1), it implies no difference or little difference in risk (incidence in each group is the equivalent). RR greater than one suggests an increased risk of the outcome in the exposed group. RR lesser than one suggests a reduced risk in the exposed group. Sub group analysis was conducted based on years of follow up (less than 10 years versus more than 10 years). Due to the high heterogeneity in the results of meta-analysis, power analysis was used to estimate the power of effect sizes. All analyzes were performed using CMA statistical software version 2.

## Result

### Study selection

**Figure 1:**
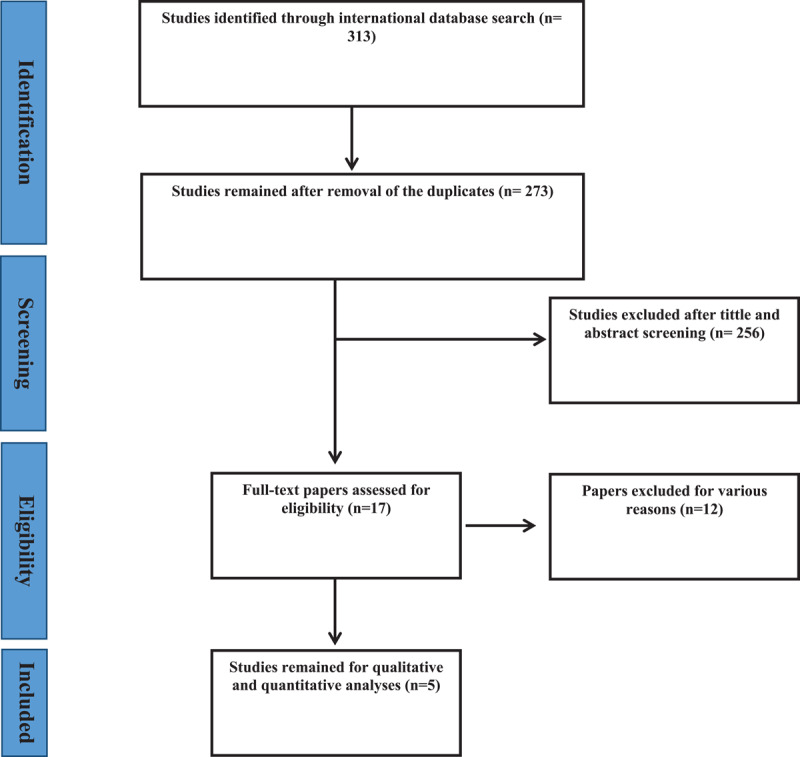
Flowchart of the included studies in the systematic review.

After searching the databases, 313 articles were found. After eliminating duplicates, 273 articles entered the title and abstract review phase. The titles and abstracts of the studies were reviewed, and 17 articles were entered into the next phase, in which the full text of the articles was reviewed, and five articles were entered into the final analysis. During the screening stage of the studies, studies could have been excluded for multiple reasons, including unrelated subject matter [10] and an unrelated study population [2]. Flowchart of the included studies is shown in Figure 1.

### Study characteristics

The articles in our analysis included 3.779 million people from 7 countries around the world, including Sweden, Norway, Netherland, Austria, Denmark, the USA, and Italy. The results of the fourteen cohorts were used in this meta-analysis. The characteristics of the included studies are listed in Table 1.

**Table 1:** Basic information of included studies.


ORDER	AUTHOR (YEAR)	LOCATION	TIME PERIOD	SAMPLE SIZE	GENDER	MEAN OF AGE	TUMOR BY MALIGNANCY	BRAIN TUMOR (n)	M ± SD pm_2.5_(μg/m^3^)	M ± SD pm_10_(μg/m^3^)	M ± SD NO_2_(μg/m^3^)	M ± SD O3(μg/m^3^)	M ± SD SO2(μg/m^3^)	M ± SD CO(μg/m^3^)

1	Andersen (2018)	Sweden	1992–96	25 600	male/female	45.9	63	0	NR	NR	5.3 (2.5)	NR	NR	NR

		Norway	2000–01	21 363	male/female	48.2	39	39	8.9 (1.3)	13.5 (3.1)	NR	NR	NR	NR

		Sweden	1992–2002	22 036	male/female	56.5	37	NR	7.1 (1.3)	14.7 (4.1)	10.8 (4.6)	NR	NR	NR

		Denmark	1993–97	38 064	male/female	56.8	200	200	11.3 (0.8)	17.2 (1.9)	16.5 (7.0)	NR	NR	NR

		Netherland	1993–97	36 505	male/female	50.3	64	64	16.9 (0.6)	25.4 (1.5)	25.2 (6.2)	NR	NR	NR

		Austria	1985–2005	131 907	male/female	41.3	NR	176	13.6 (1.2)	20.7 (2.4)	20.0 (5.5)	NR	NR	NR

		Italy	1993–97	11 893	male/female	51.6	35	34			43.4 (17.3)	NR	NR	NR

		Italy (Turin)	1993–2008	8774	male/female	50.3	28	28	30.2 (1.6)	46.6 (4.1)	53.0 (10.3)	NR	NR	NR

2	Bräuner (2013)	Denmark	1993–1997	51674	male/female	56.1	NR	121	NR	NR	22	NR	NR	NR

3	McKean (2009)	United States	1979–1983	383,620	male/female	30≤	NR	783	21.1(4.6)	NR	NR	NR	NR	NR

		United States	1999–2000	533,960	male/female	30≤	NR	1,084	14.0 (3.0)	NR	NR	NR	NR	NR

		United States	1982–1998	443,765	male/female	30≤	NR	936	NR	28.8(5.9)	NR	NR	NR	NR

		United States	1982–1999	576,315	male/female	30≤	NR	1,170	NR	NR	NR	NR	NR	NR

		United States	1982–2000	527,123	male/female	30≤	NR	1,089	NR	NR	21.3(7.1)	NR	NR	NR

		United States	1982–2001	572,829	male/female	30≤	NR	1,175	NR	NR	NR	NR	NR	1.1(0.4)

		United States	1982–2002	560,662	male/female	30≤	NR	1,135	NR	NR	NR	45.5(7.3)	NR	NR

		United States	1982–1998	560,000	male/female	30≤	NR	1,217	NR	NR	NR	59.7(12.8)	NR	NR

4	Raaschou (2011)	Denmark	1995–2006	54304	male/female	20.50	NR	NR	NR	28/4			NR	NR

5	Valberg (2010)	United States	2002–2006	NR	male/female	NR	NR	24.60	r = –0.06 & p-value = 0.26		r = –0.24 & p-value = 0.0015	r = 0.15 & p-value = 0.0013	r = –0.02 & p-value = 0.79	r = –0.28 & p-value = 0.0002


### Results of quality assessment

The results of the quality evaluation of the articles are shown in appendix I. According to our review using relevant checklists, all of the five studies were of good quality.

Egger test and funnel plots were used to investigate the publication bias in the results. The results of the publication bias of the study indicated the absence of bias in the examined results (p = 0.461) Figure 6.

### Results of heterogeneity

According to the results, there was no high heterogeneity in most selected studies. Random effect model was used with the inverse variance method for our results.

### Results of the meta-analysis

**Figure 2:**
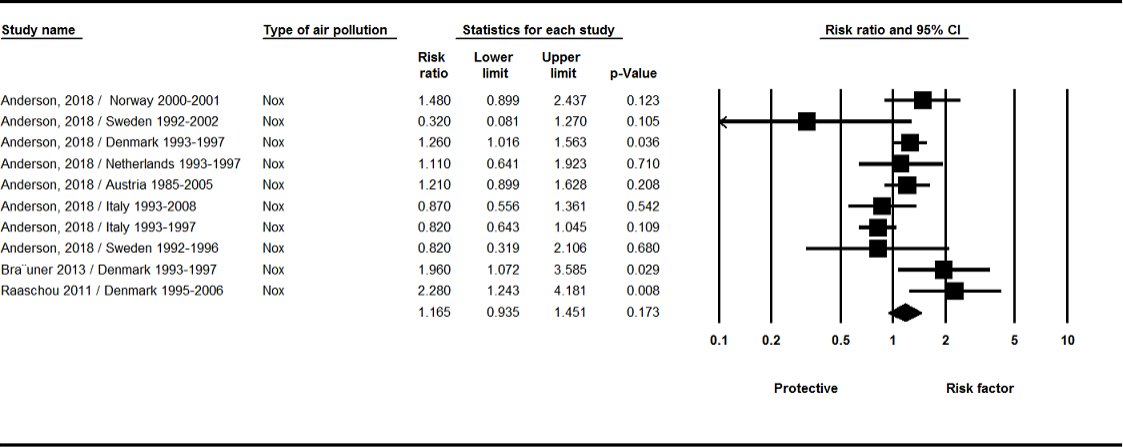
Results of the NO_x_ exposure and incidence of brain tumors.

The results of the meta-analysis are shown based on the type of air pollution index. Five studies for use were identified in our meta-analysis. All studies were of good quality, as assessed by our modified quality assessment checklist. The sample size in the analyzed articles was 3.779 million people from 14 cohorts study. As observed in Figure 2, there was a relationship between NOx index and brain tumor incidence, but this correlation was not significant (RR = 1.16, 95% CI 0.93–1.45, P = 0.173; Q-value = 22.33, df = 9; I^2^ = 59.69 %, P = 0.008).

**Figure 3:**
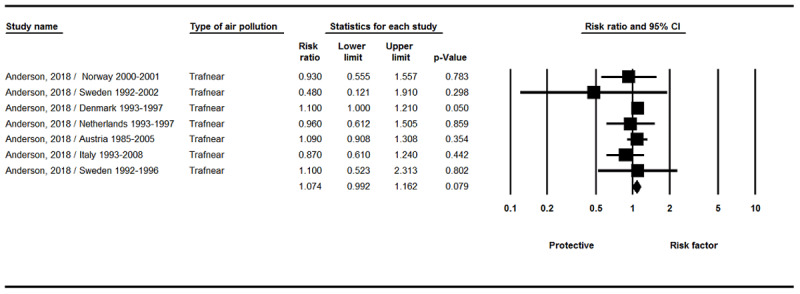
Results of the Trafnear exposure and incidence of brain tumors.

According to Figure 3, there was a correlation between Trafnear (Close to traffic) index and brain tumor incidence, but this correlation was not statistically significant (RR = 1.07, 95% CI 0.99–1.16, P = 0.079; Q-value = 3.47, df = 6; I^2^ = 0.0 %, P = 0.748).

**Figure 4:**
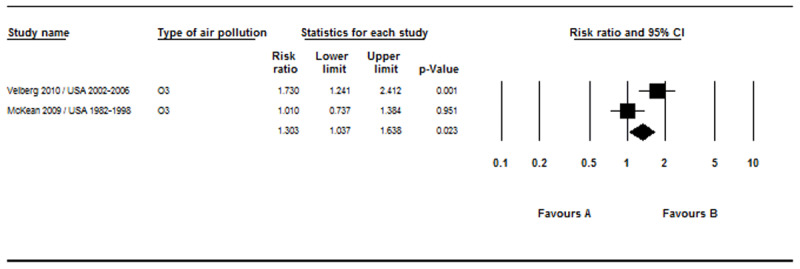
Results of the O_3_ exposure and incidence of brain tumors.

The results displayed in Figure 4 showed a significant relationship between O_3_ index and brain tumor incidence (RR = 1.30, 95% CI 1.03–1.63, P = 0.023, Q-value = 5.30, df = 6; I^2^ = 81.14 %, P = 0.021).

**Figure 5:**
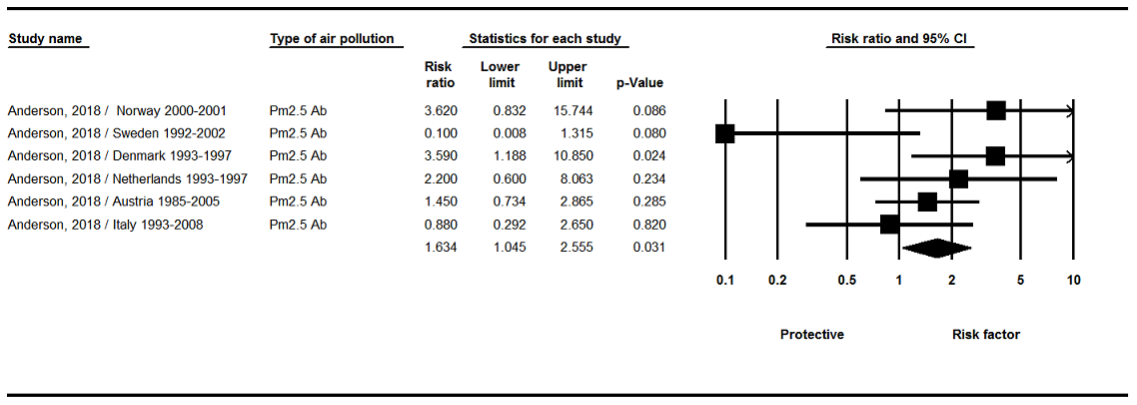
Results of the PM_2.5_ Absorbance exposure and incidence of brain tumors.

The results displayed in Figure 5 showed a significant relationship between the PM_2.5_ Absorbance index and the incidence of brain tumors (RR = 1.63, 95% CI 1.04–2.55, P = 0.031, Q-value = 9.11, df = 5; I^2^ = 45.15 %, P = 0.105).

**Figure 6:**
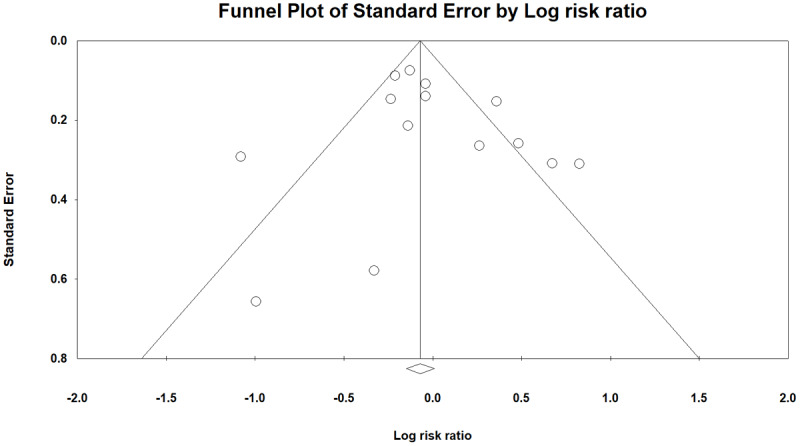
Funnel plot for assessment of publication bias.

Based on the results displayed in supplementary 1, there was no significant relationship between SO_2_ index and brain tumor incidence (RR = 0.83, 95% CI 0.69–1.00, P = 0.050, Q-value = 0.20, df = 1; I^2^ = 0.0 %, P = 0.647).

According to the results, displayed in supplementary 2, no significant relationship was found between CO index and brain tumor incidence (RR = 0.54, 95% CI 0.23–1.27, P = 0.164, Q-value = 8.13, df = 1; I^2^ = 87.71 %, P = 0.004).

As observed in supplementary 3, no significant relationship was found between NO_2_ index and brain tumor incidence (RR = 0.93, 95% CI 0.77–1.14, P = 0.530, Q-value = 27.24, df = 9; I^2^ = 67.01 %, P = 0.001).

According to the results supplementary 4, no significant relationship was observed between PM_10_ index and brain tumor incidence (RR = 0.93, 95% CI 0.83–1.04, P = 0.226, Q-value = 6.86, df = 6; I^2^ = 12.54 %, P = 0.334).

Results, displayed in supplementary 5, showed no significant relationship between the PM_2.5_ index and brain tumor incidence. (RR = 0.90, 95% CI 0.80–1.00, P = 0.064, Q-value = 5.77, df = 8; I^2^ = 0.0 %, P = 0.673).

### Sub-group analysis

The result of sub-group analysis based on years of follow up (less than 10 years versus more than 10 years) show that in Table 2.

**Table 2:** Sub-group analysis based on years of follow-up.


TYPE OF AIR POLLUTION	LESS THAN TEN YEARS FOLLOW-UP	P-VALUE	MORE THAN TEN YEARS FOLLOW-UP	P-VALUE	OVERALL	P-VALUE

NOx	1.10 (0.96–1.27)	0.152	1.17 (0.93–1.46)	0.169	1.16 (0.93–1.45)	0.173

Trafnear	1.08 (0.99–1.19)	0.069	1.02 (0.87–1.20)	0.727	1.07 (0.99–1.16)	0.079

PM_2.5_ Absorbance	3.08 (1.48–6.39)	0.003	1.11 (0.63–1.96)	0.699	1.63 (1.04–2.55)	0.031

NO_2_	1.02 (0.86–1.21)	0.758	0.88 (0.81–0.95)	0.002	0.93 (0.77–1.14)	0.530

PM10	1.05 (0.58–1.93)	0.853	0.93 (0.83–1.04)	0.205	0.93 (0.83–1.04)	0.226

PM_2.5_	0.89 (0.80–1.01)	0.066	0.93 (0.55–1.56)	0.788	0.90 (0.80–1.00)	0.064


### Power analysis

According to the results of power analysis, due to the large sample size among included studies, the values of power (1-β error probability) are close to one in all air pollution types. The results of power analysis presented in Table 3.

**Table 3:** Result of power analysis.


AIR POLLUTANT	I^2^ (%)	HETEROGENEITY	(1-β ERROR PROBABILITY)

NOx	0.0	No heterogeneity	1.0

Trafnear	81.1	High heterogeneity	1.0

O3	45.1	Low heterogeneity	1.0

PM2.5	84.0	High heterogeneity	1.0


## Discussion

In this study, the relationship between short and long term exposure to standard pollutants (including SO_2_, CO, NO_2_, PM_10_, O_3_, and PM_2.5_ Absorbance, NOx, Trafnear, and PM_2.5_) and brain tumors were reviewed. The results revealed that there was a relationship between exposure to NO_x_ index (Figure 2) and proximity to Trafnear (Figure 3) and brain tumor incidence, but this association was not significant. There was also no correlation between exposure to certain pollutants (SO_2_, CO, NO_2_, PM_10_, PM_2.5_) and brain tumor incidence (supplementary 1–5).

Epidemiological studies have recently examined the effect of exposure to air pollution and its impact on the central nervous system. Specifically, the associations between air pollution and dementia, alzheimer’s disease, parkinson’s disease, breast cancer, and stroke have been investigated, but these findings were inconclusive [29,30,31,32]. However, only a few studies have been carried to determine the relationship between exposure to air pollutants and the incidence of brain cancer. Although the number of studies in this field is scarce, these studies are mainly designed as cohorts and in developed countries. Moreover, although air pollution is mostly controlled in these countries, these pollutants have been thoroughly investigated. Consequently, there is a lack of information related to developing and underdeveloped countries.

Traffic can be a significant source of NO_2_ and CO, as well as an indispensable source of PM_2.5_ in urban areas [33]. The results of this study showed that exposure to these pollutants might have adverse effects on the central nervous system and the development of brain tumors. People in urban cities and near busy main streets appear to be the most affected. The results of Anderson et al. (2017) showed that there was a positive and weak correlation between brain cancer incidence and NOx exposure, which was not statistically significant [23]. Whereas, the results in Denmark (2011) noted that NOx exposure was significantly correlated with brain tumor incidence [34].

The results of our meta-analysis clarified that there was a statistically significant correlation between exposure to O_3_ and PM_2.5_ absorbance, (Figures 4 and 5), and brain tumors. Ozone induces a variety of poisons in humans and laboratory animals at the same concentrations found in many urban areas. These effects include morphological, functional, immunological, and biochemical changes [35]. Ozone is a robust oxidant that acts as the electron acceptor for other molecules. Typically, there is a large amount of unsaturated fatty acids in the surface of the respiratory tract and cell membranes that are under a liquid layer. The double bonds in these fatty acids are unstable. By affecting and reacting with these bonds, O_3_ produces lipohydroperoxides, aldehydes, and hydrogen peroxide, which may lead to the production of lipid radicals and automatic oxidation of cell membranes and macromolecules [35,36]. Inhalation of O_3_ pollutants may result in the release of local inflammatory mediators of the lung. Chronic lung inflammation can lead to systemic inflammation that affects the blood vessels. Cytokine secretion from systemic inflammation, including interleukins (IL-6, IL-8) and tumor necrosis factor (TNF-ɑ), that can cross the blood-brain barrier may lead to activation of microglia [37]. These active microglia may induce a tumor microenvironment. Animal studies have shown that exposure to O_3_ has been associated with cumulative rodent brain injury, and its inhalation appears to disrupt dopamine neurons in mice [29,38].

PM_2.5_ absorbance indicates black carbon aerosol due to incomplete combustion of fossil fuels in motor vehicles, especially in areas near traffic, and usually refers to elemental carbon in particulate matter PM_2.5_ [35,39].These particles may be the best indicator for proving traffic-related particles in the ultra fine particle (UFP) size range. The UFP can cross the red blood cell membrane instantly and easily [40]. There are many concerns about the central nervous system due to UFPs because, in vitro, animal studies have shown that inhaled UFPs can enter the brain by crossing the blood-brain barrier or directly through the nasal passages and olfactory neurons and accumulate in the brain [19,23,41]. These particles have a significant surface-to-volume ratio and are not bound by membrane organelles. This empowers them to interact directly with intracellular proteins, organelles, or DNA. Microscopic particles may reach specific organelles such as mitochondria, lysosomes, and nuclei. These particles in these organelles can produce sequential oxidation in the membranes. They may also release inflammatory mediators and cytokines from the cell, resulting in inflammation, oxidative stress, and DNA damage [40].

According to the results of previous studies, PM_2.5_ can cause brain-blood barrier disturbance by causing oxidative stress and neuritis. In a study reporting long-term exposure to PM_2.5_ and its association with stroke, the results manifested that increased exposure to this pollutant heightened the risk of stroke (1.12, 95% CI 1.04–1.21), and mortality caused by stroke (1.26, 95% CI 1.01–1.51) [42]. Additionally, Fu et al. investigated the relationship between short and long term exposure to PM_2.5_ and its impact on the risks of stroke and observed that short and long term exposure was associated with increased risk and mortality from stroke [43].

Experiments on animals have revealed that inhaled particles penetrate the olfactory bulb and reach the brain through the nasal cavity and specific pathways. NOx can damage neurons through the bulb or the olfactory bulb, the nasal epithelium, and the lungs by propagating, trapping, and transporting red blood cells throughout the cerebral-blood barrier pathway. NOx and cytokines released by pulmonary inflammation activate microglia, thereby provoking neuronal nitric oxide synthesis [44,45].

The McKean-Cowdin et al. cohort study showed that there was no association between increased risk of brain cancer mortality and exposure to conventional air pollutants. However, with increased exposure to sulfur dioxide, nitrogen dioxide, and carbon monoxide, there was an unforeseen risk reduction for the group. Relative risks (RR) were obtained by increasing ten ppb concentrations of sulfur dioxide or nitrogen dioxide or by increasing one ppb concentration of carbon monoxide [37].

In the present work, the design of the studies was mainly cohort. This, of course, adds to the richness of this study, but there are weaknesses, such as the limited number of studies, which are mostly related to developed and developing countries. In these articles, the most significant people with exposure to standard pollutants have been studied, but the structure and constituents of airborne particulate matter (PM_10_, PM_2.5_) have not been chemically and biologically studied.

## Conclusion

The results of this systematic review and meta-analysis, which is based on long-term exposure to ambient air pollution in adults and the incidence of a brain tumor, explicated that exposure to ozone, PM_2.5_ absorbance, proximity to traffic and NOx incidence index had the most relevant impact. The effects of ozone and PM_2.5_ absorbance were similarly statistically significant. However, no association was detected between other air pollutants and brain tumor incidence.

## Additional Files

The additional files for this article can be found as follows:

10.5334/aogh.3889.s1 Supplementary File 1. Appendix 1- Quality Assessment of included studies.

10.5334/aogh.3889.s2 Supplementary File 2. Supplementary figures 1 to 5.
