# Supplementary material for: The Relationship between Air Pollution and Brain Cancer: A Systematic Review and Meta-Analysis
Source: Ann Glob Health. 2023 Jun 23;89(1):45. doi: 10.5334/aogh.3889 (PMC10289053; doi:10.5334/aogh.3889)
Supplement: Supplementary File 1. — Appendix 1- Quality Assessment of included studies. [file agh-89-1-3889-s1.pdf]

Appendix 1- Quality Assessment of included studies

Author (year)	Selection	Comparability	Outcome	Total	Quality
Andersen & et al (2018)	3	2	3	8	Good
Bra¨uner (2013)	2	2	2	6	Good
McKean (2009)	3	2	2	7	Good
Raaschou (2011)	3	2	2	7	Good
Valberg (2010)	1	2	3	6	Good
