# Supplementary material for: The Relationship between Air Pollution and Brain Cancer: A Systematic Review and Meta-Analysis
Source: Ann Glob Health. 2023 Jun 23;89(1):45. doi: 10.5334/aogh.3889 (PMC10289053; doi:10.5334/aogh.3889)
Supplement: Supplementary File 2. — Supplementary figures 1 to 5. [file agh-89-1-3889-s2.pdf]

Study name	Type of air pollution	Risk ratio	Lower limit	Upper limit	p-Value
Velberg 2010 / USA 2002-2006	So2	0.930	0.563	1.537	0.777
McKean 2009 / USA 1982-1998	So2	0.820	0.675	0.997	0.046
		0.834	0.695	1.000	0.050

Statistics for each study

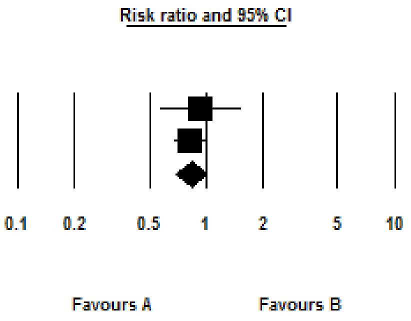

Supplementary figure 1.

Results of the SO_2 exposure and incidence of brain tumors

Study name	Type of air pollution	Risk ratio	Lower limit	Upper limit	p-Value
		Statistics for each study			
Velberg 2010 / USA 2002-2006	Co	0.340	0.192	0.602	0.000
McKean 2009 / USA 1982-1998	Co	0.810	0.682	0.962	0.017
		0.549	0.235	1.279	0.164

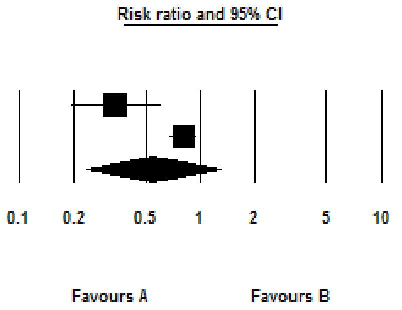

Supplementary figure 2.

Results of the CO exposure and incidence of brain tumors

Study name	Type of air pollution	Risk ratio	Lower limit	Upper limit	p-Value
		Statistics for each study			
Anderson, 2018 / Norway 2000-2001	No2	1.620	0.976	2.689	0.062
Anderson, 2018 / Sweden 1992-2002	No2	0.370	0.102	1.339	0.130
Anderson, 2018 / Denmark 1993-1997	No2	1.430	1.060	1.930	0.019
Anderson, 2018 / Netherlands 1993-1997	No2	1.300	0.774	2.182	0.321
Anderson, 2018 / Austria 1985-2005	No2	0.960	0.731	1.261	0.769
Anderson, 2018 / Italy 1993-2008	No2	0.870	0.572	1.324	0.516
Velberg 2010 / USA 2002-2006	No2	0.400	0.226	0.707	0.002
Anderson, 2018 / Italy 1993-1997	No2	0.720	0.232	2.237	0.570
Anderson, 2018 / Sweden 1992-1996	No2	0.790	0.592	1.054	0.109
McKean 2009 / USA 1982-1998	No2	0.880	0.808	0.958	0.003
		0.938	0.770	1.144	0.530

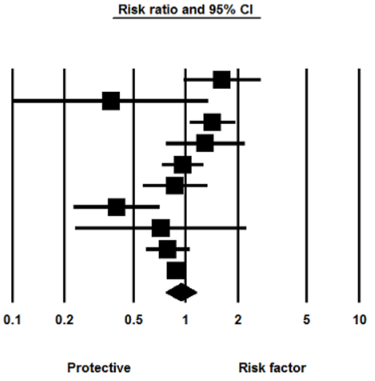

Supplementary figure 3.

Results of the NO_2 exposure and incidence of brain tumors

Study name	Type of air pollution	Statistics for each study			
		Risk ratio	Lower limit	Upper limit	p-Value
Anderson, 2018 / Norway 2000-2001	Pm10	0.880	0.292	2.650	0.820
Anderson, 2018 / Sweden 1992-2002	Pm10	0.580	0.122	2.751	0.493
Anderson, 2018 / Denmark 1993-1997	Pm10	0.690	0.288	1.656	0.406
Anderson, 2018 / Netherlands 1993-1997	Pm10	3.200	0.919	11.145	0.068
Anderson, 2018 / Austria 1985-2005	Pm10	3.180	0.420	24.059	0.263
Anderson, 2018 / Italy 1993-2008	Pm10	1.240	0.670	2.295	0.494
McKean 2009 / USA 1982-1998	Pm10	0.920	0.821	1.031	0.152
		0.934	0.837	1.043	0.226

Risk ratio and 95% CI

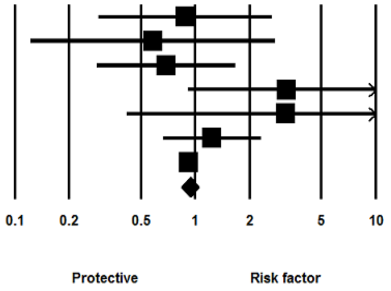

Supplementary figure 4.

Results of the Pm10 exposure and incidence of brain tumors

Study name	Type of air pollution	Statistics for each study				Risk ratio and 95% CI
		Risk ratio	Lower limit	Upper limit	p-Value	
Anderson, 2018 / Norway 2000-2001	Pm2.5	0.610	0.118	3.150	0.555	
Anderson, 2018 / Sweden 1992-2002	Pm2.5	0.300	0.080	1.122	0.074	
Anderson, 2018 / Denmark 1993-1997	Pm2.5	1.410	0.321	6.191	0.649	
Anderson, 2018 / Netherlands 1993-1997	Pm2.5	2.980	0.280	31.703	0.365	
Anderson, 2018 / Austria 1985-2005	Pm2.5	1.170	0.635	2.154	0.614	
Anderson, 2018 / Italy 1993-2008	Pm2.5	1.010	0.238	4.285	0.989	
McKean 2009 / USA 1979-1983	Pm2.5	0.880	0.760	1.019	0.089	
McKean 2009 / USA 1999-2000	Pm2.5	0.960	0.777	1.186	0.705	
Velberg 2010 / USA 2002-2006	Pm2.5	0.800	0.549	1.167	0.247	
		0.900	0.805	1.006	0.064	

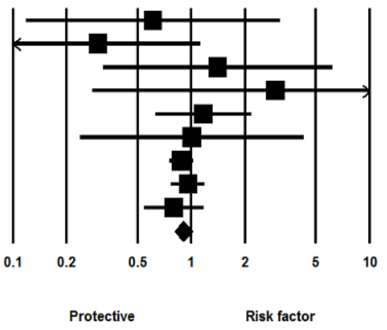

Supplementary figure 5.

Results for the Pm2.5 exposure and brain tumor incidence
